# Preliminary Programme of the Medical Association of Georgia

**Published:** 1892-04

**Authors:** G. W. Mulligan, Dan H. Howell

**Affiliations:** President; Secretary


### PRELIMINARY PROGRAMME OF THE MEDICAL ASSOCIATION OF GEORGIA.

TO BE HELD IN COLUMBUS, GA., APRIL 20TH, 21ST AND 22D, 1892.

The President's Annual Address—G. W. Mulligan, M. D., Washington.

Cough—Some of its Causes and Treatment—Chas. D. Roy, A. B., M. D., Atlanta.

So-Called Typho-Malarial Fever—W. P. Williams, M. D., Waycross.

Preliminary Observation on the Behavior of Iodine in the Presence of Camphor, Menthol, Thymol, etc.—R. J. Nunn, M. D., Savannah.

Some Observations upon Cataract Operations and After Treatment—A. W. Calhoun, M. D., Atlanta.

Remittent Fever—A. C. Blain, M. D., Brunswick.

Extirpation of Rectum for Carcinoma—J. McF. Gaston, M. D., Atlanta.

Some of the Fads and Fancies of the Medical Profession—J. C. LeHardy, M. D., Savannah.

Treatment of Pneumonia, with Report of Cases—H. Perdue, M. D., Barnesville.

How shall we Manage the Uterus after Abortion?—K. P. Moore, M. D., Macon.

Plaster of Paris in Surgery—W. F. Westmoreland, M. D., Atlanta.

Report of Surgical Cases from my Note Book—J. B. Hinkle, M. D., Americus.

What is Gynæcology?—R. R. Kime, M. D., Atlanta.

A Case of Ovarian Cysts—J. M. Spence, M. D., Waresboro.

Some Remarks on Tonsil Excisions, with Presentation and Description of a New Instrument—A. G. Hobbs, M. D., Atlanta.

How Best to Conduct Labor to Prevent Injuries to the Os Uteri and Perineum—A. W. Griggs, M. D., West Point.

Gunshot Wounds of Eye—Unusual Results—Geo. A. Wilcox, M. D., Augusta.

The Treatment of Abortion and some of the Complications—Walter A. Crow, M. D., Atlanta.

Report of Case of Catalepsy and Treatment—Albert Sydney Johnson, M. D., Bowman.

Typhlitis and Report of Case—S. M. Mathews, M. D., Quitman.

The Relations and Dependencies Existing between the Specialist and General Practitioner of Medicine and Surgery—J. W. Griggs, M. D., West Point.

The Relation between Skin Diseases and the General Health—M. B. Hutchins, M. D., Atlanta.

Treatment of Hæmorrhoids by Carbolic Acid Injection—J. W. Hallum, M. D., Carrollton.

Hemeralopia or Night Blindness—S. Latimer Phillips, M. D., Savannah.

A Combination of Carbolic Acid and Camphor, as an Antiseptic—Wm. Perrin Nicolson, M. D., Atlanta.

Chorea—Hugh Hagan, M. D., Atlanta.

Antiseptic Surgery—Ralph E. Smith, M. D., Atlanta.

Radical Surgery the Best Surgery in the Treatment of Extensive Lacerated and Contused Wounds of the Extremities—E. H. Richardson, M. D., Atlanta.

Typho-Malarial Fever—J. W. Duncan, M. D., Atlanta.

The Action of Fibroid Tumors after the Menopause—Virgil O. Hardon, M D., Atlanta.

Intestinal Obstruction, Varieties, Diagnosis and Treatment.—J. B. Hinkle, M. D., Americus.

Report of Perineal Sections for Sticture, Stone in Bladder and Cystitis—Floyd W. McRae, M. D., Atlanta.

Suprapubic Lithotomy with Report of Cases—W. S. Elkin, M. D., Atlanta.

Typhlitis—William O'Daniel, M. D., Bullard.

G. W. MULLIGAN, M. D., President.

DAN H. HOWELL, M. D., Secretary.

